# Development and validation of a diagnostic model to differentiate spinal tuberculosis from pyogenic spondylitis by combining multiple machine learning algorithms

**DOI:** 10.17305/bb.2023.9663

**Published:** 2024-04-01

**Authors:** Chengqian Huang, Jing Zhuo, Chong Liu, Shaofeng Wu, Jichong Zhu, Tianyou Chen, Bin Zhang, Sitan Feng, Chenxing Zhou, Zequn Wang, Shengsheng Huang, Liyi Chen, Xinli Zhan

**Affiliations:** 1 Department of Spine and Osteopathy Ward, The First Affiliated Hospital of Guangxi Medical University, Nanning, China; 2 Surgical Operation Department, Baise People’s Hospital, Affiliated Southwest Hospital of Youjiang Medical University for Nationalities, Baise, China

**Keywords:** Spinal tuberculosis (STB), pyogenic spondylitis (PS), machine learning (ML), diagnostic model, nomogram

## Abstract

This study focused on the development and validation of a diagnostic model to differentiate between spinal tuberculosis (STB) and pyogenic spondylitis (PS). We analyzed a total of 387 confirmed cases, out of which 241 were diagnosed with STB and 146 were diagnosed with PS. These cases were randomly divided into a training group (*n* ═ 271) and a validation group (*n* ═ 116). Within the training group, four machine learning (ML) algorithms (least absolute shrinkage and selection operator [LASSO], logistic regression analysis, random forest, and support vector machine recursive feature elimination [SVM-RFE]) were employed to identify distinctive variables. These specific variables were then utilized to construct a diagnostic model. The model’s performance was subsequently assessed using the receiver operating characteristic (ROC) curves and the calibration curves. Finally, internal validation of the model was undertaken in the validation group. Our findings indicate that PS patients had an average platelet-to-neutrophil ratio (PNR) of 277.86, which was significantly higher than the STB patients’ average of 69.88. The average age of PS patients was 54.71 years, older than the 48 years recorded for STB patients. Notably, the neutrophil-to-lymphocyte ratio (NLR) was higher in PS patients at 6.15, compared to the 3.46 NLR in STB patients. Additionally, the platelet volume distribution width (PDW) in PS patients was 0.2, compared to 0.15 in STB patients. Conversely, the mean platelet volume (MPV) was lower in PS patients at an average of 4.41, whereas STB patients averaged 8.31. Hemoglobin (HGB) levels were lower in PS patients at an average of 113.31 compared to STB patients’ average of 121.64. Furthermore, the average red blood cell (RBC) count was 4.26 in PS patients, which was less than the 4.58 average observed in STB patients. After evaluation, seven key factors were identified using the four ML algorithms, forming the basis of our diagnostic model. The training and validation groups yielded area under the curve (AUC) values of 0.841 and 0.83, respectively. The calibration curves demonstrated a high alignment between the nomogram-predicted values and the actual measurements. The decision curve indicated optimal model performance with a threshold set between 2% and 88%. In conclusion, our model offers healthcare practitioners a reliable tool to efficiently and precisely differentiate between STB and PS, thereby facilitating swift and accurate diagnoses.

## Introduction

Spinal tuberculosis (STB) is a prevalent form of secondary tuberculosis [1]. This infectious disease, caused by *Mycobacterium tuberculosis,* accounts for approximately half of all cases of bone tuberculosis [2, 3]. Every year, 1.3 million people succumb to tuberculosis (TB), with STB being the most common musculoskeletal manifestation, accounting for approximately 1% to 2% of all TB cases [4]. Individuals with STB typically present with insidious onset back pain that may gradually intensify [5]. Patients may experience night sweats, low-grade fever, and weight loss. As the condition progresses, neurological symptoms may arise, resulting in deficits, such as weakness, numbness, and paralysis.

Certain atypical STB cases can be easily mistaken for pyogenic spondylitis (PS) due to the lack of specific clinical manifestations and inconclusive imaging examinations [6]. PS, usually caused by bacterial infections, most frequently by *Staphylococcus aureus*, typically presents with acute, severe back pain accompanied by localized tenderness and swelling. Systemic symptoms, such as fever and chills, are common. While neurological symptoms can manifest in both conditions, they tend to be acute and severe in PS, contrasting with the more gradual progression observed in STB. Differentiating between STB and PS poses a challenge because of their overlapping clinical features and similar radiological findings. Both conditions can cause back pain, fever, and neurological symptoms. Adding to the complexity, initial imaging modalities, such as X-rays and MRI, may not always provide a definitive differentiation. In numerous instances, a detailed history, microbiological tests, and occasionally even a biopsy may be required for a precise diagnosis [7]. Misdiagnosing can result in therapeutic delays, underscoring the critical importance of correctly differentiating between these two conditions.

Machine learning (ML), a technology originating from computer science, facilitates efficient data processing through rapid computational methods [8]. It is currently being extensively applied to analyze clinical data [9–11]. For instance, Zhu et al. [12] utilized ML methods to construct diagnostic models for patients with ankylosing spondylitis, while Zhou et al. [13] employed ML algorithms to classify patients with cervical spondylotic myelopathy and assess the post-surgical therapeutic effects for each category.

Recognizing the potential of ML in processing vast amounts of clinical data, we intended to use ML methods to process STB-related data. Given that many basic healthcare facilities do not have access to specialized TB diagnostic tests and pathological examinations, distinguishing between STB and PS is often challenging. Such delays in diagnosis and subsequent treatment can be detrimental. Our objective is to utilize commonly available diagnostic tests to develop predictive models for these conditions. The primary focus of this study is to analyze the differences in blood samples between STB and PS patients. By utilizing ML methods, we aim to construct a diagnostic model that will enhance diagnostic efficiency for clinicians, thereby facilitating the initiation of early systemic treatments for affected individuals.

## Materials and methods

### Patients

In this study, a retrospective analysis of patients who underwent surgery for either STB or PS at the First Affiliated Hospital of Guangxi Medical University between January 2015 and May 2022, was conducted. The inclusion criteria were as follows: (1) patients who received a postoperative diagnosis of STB or PS based on pathological examination and (2) patients with available preoperative blood routine examination data and sufficient clinical information. The exclusion criteria were as follows: (1) patients with an uncertain pathological diagnosis following surgery; (2) patients diagnosed with concurrent autoimmune disorders or cancers; (3) patients with active infections in other bodily organs or sites; and (4) patients with incomplete clinical information. Data collected for statistical analysis encompassed sex, age, C-reactive protein (CRP), erythrocyte sedimentation rate (ESR), and complete blood count. The complete blood count included parameters, such as red blood cells (RBCs), white blood cells (WBCs), hemoglobin (HGB), neutrophil count (NEU), platelet count (PLT), monocyte count (MONO), lymphocyte count (LYM), eosinophil count (EOS), basophil count (BASO), mean RBC hemoglobin content (MCH), mean red blood cell volume (MCV), mean corpuscular hemoglobin concentration (MCHC), RBC volume distribution width coefficient of variation (RDWCV), mean platelet volume (MPV), platelet volume distribution width (PDW), plateletcrit (PCT), platelet-to-monocyte ratio (PMR), monocyte-to-lymphocyte ratio (MLR), neutrophil-to-lymphocyte ratio (NLR), platelet-to-lymphocyte ratio (PLR), and platelet-to-neutrophil ratio (PNR). In total, 387 patients were enrolled, out of whom 241 were diagnosed with STB and 146 were diagnosed with PS. These patients were subsequently randomized into either the training or the validation group at a ratio of 7:3 (Tables S1 and S2).

### Logistic regression analysis

Logistic regression, a statistical method designed for binary classification, was employed in this study. It models the relationship between a binary dependent variable and one or more independent variables, estimating the probability of the dependent variable falling into a specific category based on the values of the independent variables. Logistic regression aided in variable selection, with variables exhibiting a *P* value of < 0.05 being considered as predictive variables.

### A LASSO regression analysis

A LASSO regression model was developed to identify risk factors and determine optimal predictors for STB patients from a pool of variables that could potentially be collinear. The LASSO regression was conducted using the “glmnet” package in the R software [14].

### Random forest analysis

Random forest analysis was performed using the “random forest” package in R software for variable selection and evaluation of their importance. Variables received random values, and an increase in the mean squared error (MSE) after randomly altering a variable’s value indicated its importance. The metric “IncNodePurity”, which depicts the influence of a variable on the heterogeneity of observed values in the classification tree, was used to determine variable importance. The variable with the highest “IncNodePurity” value, determined through 10-fold cross-validation, was selected.

### Support vector machine

The support vector machine recursive feature elimination (SVM-RFE), a powerful ML approach, was employed and constructed using the “rms” package. Data generated underwent 10-fold cross-validation to obtain the output vector characteristic index. Subsequently, variables were ranked based on their usefulness, from the most to the least useful [15].

**Table 1:** Comparison of clinical data in the STB and PS patient groups

**Clinical factors**	**Training cohort (*n* ═ 271)**			**Validation cohort (*n* ═ 116)**		
	**STB (*n* ═ 169)**	**PS (*n* ═ 102)**	***P* value**	**STB (*n* ═ 72)**	**PS (*n* ═ 44)**	***P* value**
Age (years)	48 ± 16.78	54.71 ± 15.11	**0.001**	49.24 ± 18.39	52.77 ± 17.24	0.306
ESR (mm/hr)	42.2 ± 24.25	47.34 ± 29.48	0.139	40.15 ± 24.69	47.27 ± 25.27	0.138
Sex			0.999			0.190
Male	111 (62.4%)	67 (37.6%)		42 (57.5%)	34 (42.5%)	
Female	58 (62.4%)	35 (37.6%)		30 (69.8%)	13 (30.2%)	
CRP (mg/L)	32.06 ± 43.32	34.46 ± 40.02	0.650	25.67 ± 33.86	29.51 ± 34.69	0.558
WBC (10^9^/L)	7.23 ± 2.51	7.5 ± 3.9	0.534	7.54 ± 2.39	7.42 ± 2.05	0.790
RBC (10^12^/L)	4.58 ± 0.7	4.26 ± 0.86	**0.002**	4.56 ± 0.6	4.32 ± 0.6	**0.042**
HGB (g/dL)	121.64 ± 17.52	113.31 ± 21.29	**0.001**	122.76 ± 13.44	115.53 ± 17.9	**0.015**
PLT (10^9^/L)	288.75 ± 81.4	310.98 ± 112.3	**0.083**	304.96 ± 103.23	339.69 ± 90.75	0.069
NEU (10^9^/L)	4.69 ± 2.12	3.1 ± 3.97	**< 0.001**	4.74 ± 1.86	2.86 ± 2.35	**< 0.001**
LYM (10^9^/L)	1.62 ± 0.83	0.93 ± 0.96	**< 0.001**	1.69 ± 0.79	1.03 ± 0.98	**< 0.001**
MONO (10^9^/L)	0.64 ± 0.24	0.36 ± 0.4	**< 0.001**	0.68 ± 0.26	0.34 ± 0.35	**< 0.001**
EOS (10^9^/L)	0.25 ± 0.23	0.83 ± 0.82	**< 0.001**	0.38 ± 0.7	0.83 ± 0.78	**0.002**
BASO (10^9^/L)	0.04 ± 0.02	0.1 ± 0.12	**< 0.001**	0.04 ± 0.02	0.11 ± 0.19	**0.011**
MCV (fL)	82.02 ± 9.53	82.93 ± 10.03	0.455	82.77 ± 8.94	82.87 ± 10.06	0.954
MCH (pg)	26.83 ± 3.57	26.94 ± 3.87	0.806	27.24 ± 3.47	26.96 ± 3.9	0.683
MCHC (g/dL)	326.49 ± 11.76	324 ± 11.78	0.093	328.42 ± 11.25	324.36 ± 12.89	0.077
RDWCV	0.15 ± 0.03	0.24 ± 0.11	**< 0.001**	0.15 ± 0.02	0.24 ± 0.11	**< 0.001**
MPV (fL)	8.31 ± 1.14	4.41 ± 4.05	**< 0.001**	8.1 ± 1.06	4.3 ± 3.88	**< 0.001**
PCT	0.24 ± 0.07	0.2 ± 0.07	**< 0.001**	0.16 ± 0.02	0.21 ± 0.08	**0.011**
PDW	0.15 ± 0.02	0.2 ± 0.08	**< 0.001**	0.37 ± 0.04	3.65 ± 3.7	**0.000**
MLR	0.47 ± 0.26	0.31 ± 0.4	**0.001**	0.45 ± 0.2	0.27 ± 0.3	**0.000**
PMR	502.44 ± 201.81	85411.16 ± 431851.78	**0.050**	493.9 ± 200.43	40774.29 ± 68766.21	**0.000**
PLR	207.92 ± 91.24	1921.72 ± 2409.28	**< 0.001**	211.73 ± 109.78	3531.68 ± 10672.98	**0.045**
NLR	3.46 ± 2.38	6.15 ± 5.45	**< 0.001**	3.22 ± 1.52	8.21 ± 21.21	**0.127**
PNR	69.88 ± 26.76	277.86 ± 251.28	**< 0.001**	68.73 ± 22.38	293.1 ± 264.08	**0.000**

Data are represented as mean ± standard deviation or *n* (%). The bolded *P* values denote statistical significance (*P* < 0.05). STB: Spinal tuberculosis; PS: Pyogenic spondylitis; ESR: Erythrocyte sedimentation rate; CRP: C-reactive protein; WBC: White blood cells; RBC: Red blood cells; HGB: Hemoglobin; PLT: Platelets; NEU: Neutrophil count; LYM: Lymphocyte count; MONO: Monocyte count; EOS: Eosinophil count; BASO: Basophil count; MCV: Mean red blood cell volume; MCH: Mean RBC hemoglobin content; MCHC: Mean corpuscular hemoglobin concentration; RDWCV: RBC volume distribution width coefficient of variation; MPV: Mean platelet volume; PCT: Plateletcrit; PDW: Platelet volume distribution width; HCT: Hematocrit; MLR: Monocyte-to-lymphocyte ratio; PMR: Platelets-to-monocyte ratio; PLR: Platelets-to-lymphocyte ratio; NLR: Neutrophil-to-lymphocyte ratio; PNR: Platelets-to-neutrophil ratio.

### Intersection variable selection

These four distinct methods were utilized to screen predictive variables. Common variables were identified using a Venn diagram, from which a dynamic prediction model was constructed. The model’s performance was assessed through receiver operating characteristic (ROC) and calibration curves. Its effectiveness was further confirmed using the validation group.

### Ethical statement

Ethical approval for this study was obtained from the Ethics Committee of the First Affiliated Hospital of Guangxi Medical University (approval number: 2023-E177–01).

### Statistical analysis

Statistical analyses were performed using the R statistical software (version 4.2.1) and SPSS (version 26.0, IBM). For continuous variables, the *t*-test or Mann–Whitney *U* test was employed, while categorical variables were assessed using the chi-square test or Fisher’s exact test. Pearson’s test was used for correlation analysis of data with a normal distribution, whereas Spearman’s test was applied for data with a non-normal distribution. All continuous data were expressed as mean ± standard deviation (SD). A *P* value of < 0.05 was considered statistically significant.

## Results

### Data characteristics

**Figure 1.**
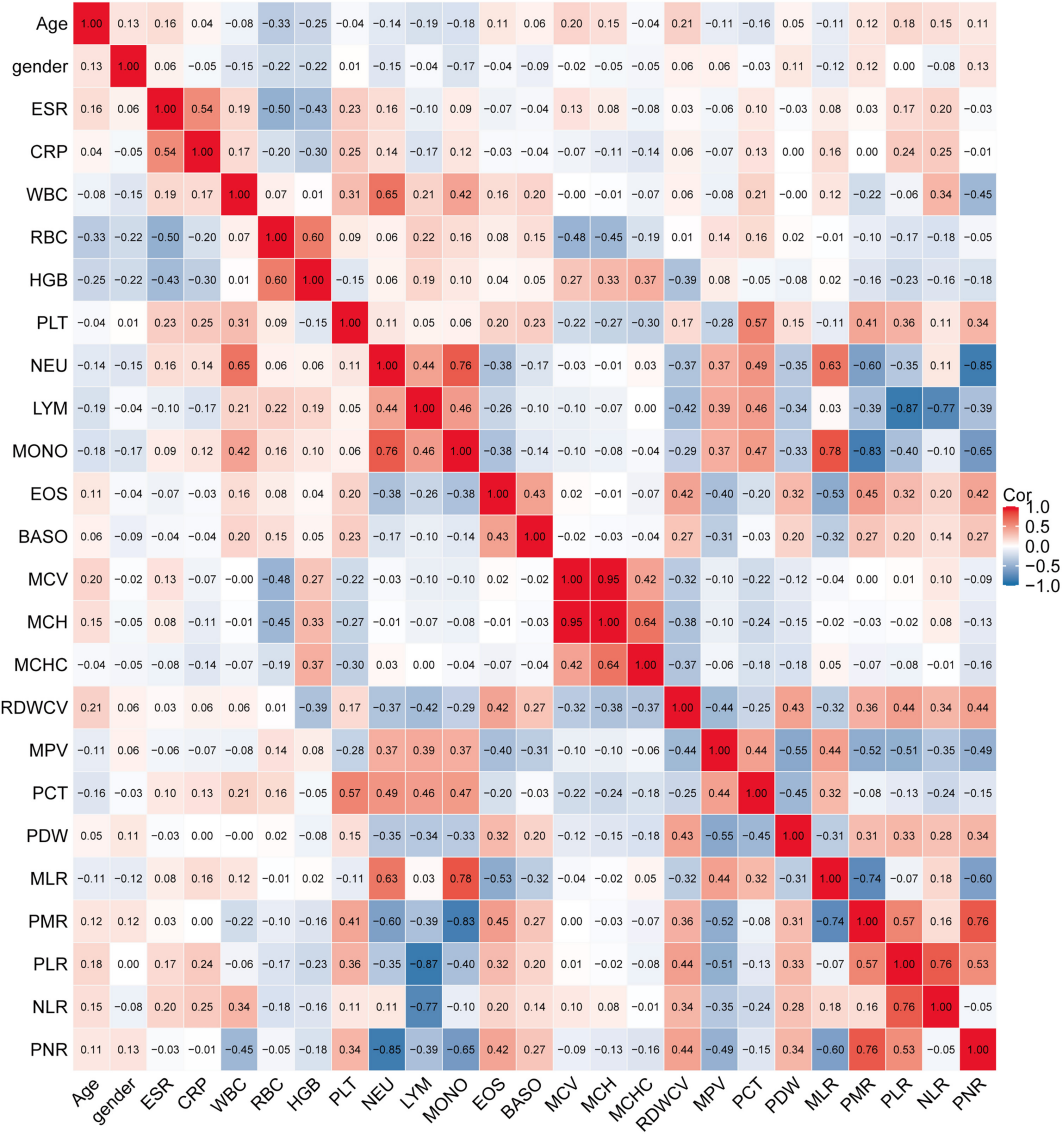
**Heatmap displaying correlations among all variables.** ESR: Erythrocyte sedimentation rate; CRP: C-reactive protein; WBC: White blood cells; RBC: Red blood cells; HGB: Hemoglobin; PLT: Platelets; NEU: Neutrophil count; LYM: Lymphocyte count; MONO: Monocyte count; EOS: Eosinophil count; BASO: Basophil count; MCV: Mean red blood cell volume; MCH: Mean RBC hemoglobin content; MCHC: Mean corpuscular hemoglobin concentration; RDWCV: RBC volume distribution width coefficient of variation; MPV: Mean platelet volume; PCT: Plateletcrit; PDW: Platelet volume distribution width; MLR: Monocyte-to-lymphocyte ratio; PMR: Platelets-to-monocyte ratio; PLR: Platelets-to-lymphocyte ratio; NLR: Neutrophil-to-lymphocyte ratio; PNR: Platelets-to-neutrophil ratio; Cor: Correlation.

A total of 387 patients who met the inclusion criteria were enrolled in the study. This comprised 241 patients with STB, accounting for 62.27% of the total, and 146 patients with PS, representing 37.73% of the cohort. The distribution features of both groups are detailed in Table 1. Our findings indicated that PS patients exhibited a notably higher PNR, averaging 277.86, compared to STB patients who averaged 69.88. PS patients also had an older average age of 54.71 years, compared to 48 years for STB patients. The NLR in PS patients was 6.15, surpassing the NLR of 3.46 observed in STB patients. Furthermore, PS patients displayed a higher PDW of 0.20 in contrast to 0.15 observed in STB patients. Conversely, the MPV was lower in PS patients, averaging 4.41, while it was 8.31 in STB patients. HGB levels in PS patients stood at an average of 113.31, whereas STB patients averaged 121.64. Moreover, RBC counts in PS patients averaged 4.26, which was lower than the 4.58 average of STB patients. Figure 1 illustrates the associations between the variables in the validation cohort. Within Figure 1, a clear positive correlation is evident between MCV and MCH, as well as between PNR and NLR. Conversely, PLR and PMR exhibit a distinct negative correlation.

### Logistic regression analysis results

The logistic regression analysis, as presented in Table 2, revealed a significant difference between the two patient groups (*P* < 0.05). The assessed variables encompassed age, RBC, HGB, NEU, LYM, MONO, EOS, BASO, RDWCV, MPV, PCT, PDW, MLR, PLR, NLR, and PNR.

**Table 2:** Logistic regression analysis results

**Variable**	**OR**	**Lower limit of 95% CI**	**Upper limit of 95% CI**	***P* value**
Age (years)	0.974	0.958	0.990	0.001
BASO	< 0.001	< 0.001	< 0.001	< 0.001
CRP	0.999	0.993	1.005	0.649
EOS	0.097	0.044	0.192	< 0.001
ESR	0.993	0.983	1.002	0.121
Sex	1.000	0.598	1.686	0.999
HGB	1.023	1.010	1.037	0.001
LYM	3.081	2.138	4.574	< 0.001
MCH	0.992	0.926	1.060	0.805
MCHC	1.018	0.997	1.041	0.094
MCV	0.990	0.965	1.016	0.454
MLR	6.707	2.570	19.669	< 0.001
MONO	22.389	8.717	62.759	< 0.001
MPV	1.698	1.476	2.037	< 0.001
NEU	1.304	1.159	1.483	< 0.001
NLR	0.814	0.742	0.883	< 0.001
PCT	8507.151	132.409	802524.327	< 0.001
PDW	< 0.001	< 0.001	< 0.001	< 0.001
PLR	0.997	0.995	0.998	0.001
PLT	0.998	0.995	1.000	0.063
PMR	1.000	0.998	1.000	0.394
PNR	0.985	0.978	0.990	< 0.001
RBC	1.734	1.243	2.462	0.002
RDWCV	< 0.001	< 0.001	< 0.001	< 0.001
WBC	0.973	0.898	1.054	0.491

OR: Odds ratio; BASO: Basophil count; CRP: C-reactive protein; EOS: Eosinophil count; ESR: Erythrocyte sedimentation rate; HGB: Hemoglobin; LYM: Lymphocyte count; MCH: Mean RBC hemoglobin content; MCHC: Mean corpuscular hemoglobin concentration; MCV: Mean red blood cell volume; MLR: Monocyte-to-lymphocyte ratio; MONO: Monocyte count; MPV: Mean platelet volume; NEU: Neutrophil count; NLR: Neutrophil-to-lymphocyte ratio; PCT: Plateletcrit; PDW: Platelet volume distribution width; PLR: Platelets-to-lymphocyte ratio; PLT: Platelets; PMR: Platelets-to-monocyte ratio; PNR: Platelets-to-neutrophil ratio; RBC: Red blood cells; RDWCV: RBC volume distribution width coefficient of variation; WBC: White blood cells.

### The LASSO analysis results

**Figure 2.**
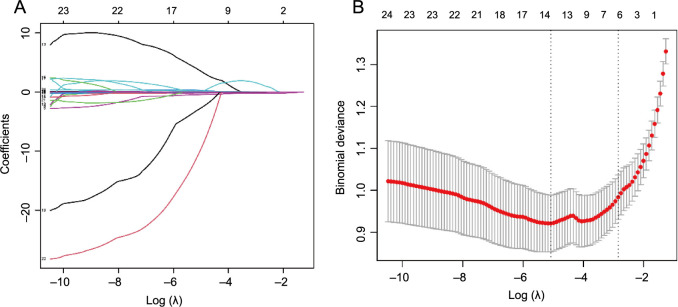
**LASSO coefficient profiles of the factors, determined by cross-validation for the optimal penalty parameter lambda.** (A) Depiction of the results of the LASSO regression analysis of dependent variables; (B) Presentation of the 13 factors that exhibited significant differences between the STB and PS patient groups. LASSO: Least absolute shrinkage and selection operator; STB: Spinal tuberculosis; PS: Pyogenic spondylitis.

The results of the LASSO analysis regarding the dependent variables are depicted in Figure 2A. Figure 2B displays the 13 factors that exhibited significant differences between the STB and PS patient groups, as identified by the LASSO regression. The factors selected through LASSO regression include age, sex, ESR, RBC, HGB, LYM, BASO, MCHC, MPV, PCT, PDW, NLR, and PNR.

### The SVM-RFE and random forest results

**Figure 3.**
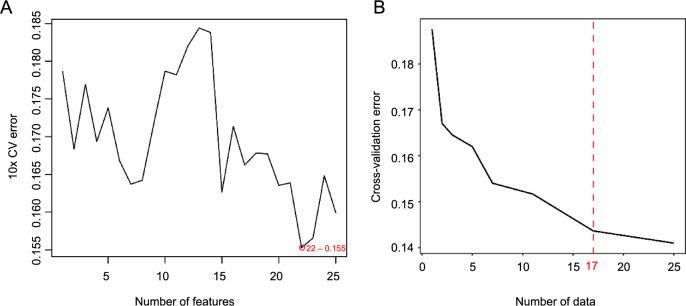
**Selection of important variables using SVM-RFE andrandom forest methods.** (A) The 22 factors identified as diagnostic models following the SVM-RFE computation; (B) The 17 factors selected as diagnostic models after calculation with the random forest method. SVM-RFE: Support vector machine recursive feature elimination; CV: Cross-validation.

As illustrated in Figure 3A, the diagnostic model identified 22 factors following computation based on the SVM-RFE algorithm, which yielded the lowest error rate. These factors, ranked by their importance as determined by SVM-RFE, encompass PNR, NLR, PCT, PDW, RBC, BASO, MCV, MCHC, MLR, age, NEU, PLT, WBC, RDWCV, sex, EOS, PLR, HGB, MONO, MCH, MPV, and CRP. These selected factors were considered to be of particular importance for the diagnosis. The factors with the highest level of importance were identified through the “IncNodePurity” random forest algorithm. As depicted in Figure 3B, the most effective regression was achieved by retaining the top 17 factors based on their importance, following the 10-fold cross-validation.

### Model development

**Figure 4.**
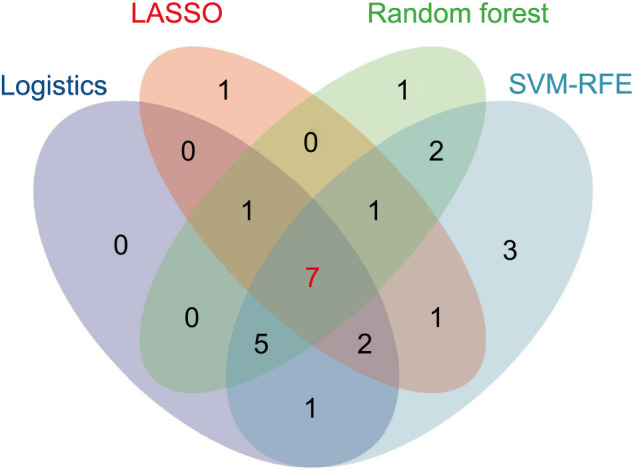
**The intersection of variables identified using logistic regression analysis, LASSO, random forest, and SVM-RFE methods.** LASSO: Least absolute shrinkage and selection operator; SVM-RFE: Support vector machine recursive feature elimination.

**Figure 5.**
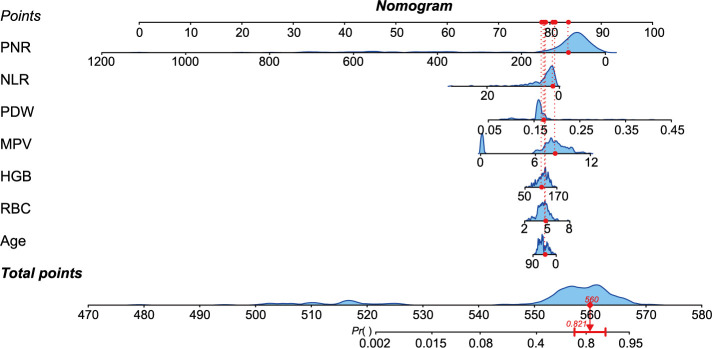
**The nomogram diagnostic model for differentiating STB from PS.** STB: Spinal tuberculosis; PS: Pyogenic spondylitis; PNR: Platelets-to-neutrophil ratio; NLR: Neutrophil-to-lymphocyte ratio; PDW: Platelet volume distribution width; MPV: Mean platelet volume; HGB: Hemoglobin; RBC: Red blood cells.

We identified the common variables from the logistic regression analysis, LASSO regression, SVM-RFE, and random forest methods, resulting in a total of seven predictors: PNR, NLR, PDW, MPV, HGB, RBC, and age. Figure 4 displays the overlap of variables identified through these four methods. Subsequently, we constructed a nomogram diagnostic model, which is presented in Figure 5.

### Model performance

**Figure 6.**
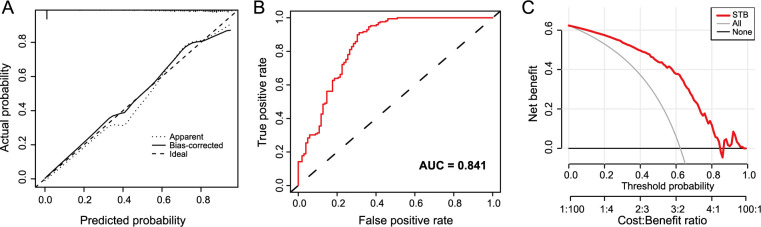
**The diagnostic performance of the nomogram prediction model in the training cohort.** (A) The calibration curve of the nomogram diagnostic model in the training cohort; (B) The ROC curves of the nomogram diagnostic model in the training cohort; (C) The decision curve analysis of the nomogram diagnostic model in the training cohort. ROC: Receiver operating characteristic; AUC: Area under the curve; STB: Spinal tuberculosis.

**Figure 7.**
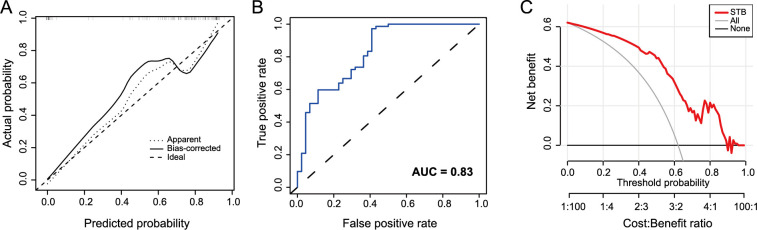
**The diagnostic performance of the nomogram prediction model in the validation cohort.** (A) The calibration curve of the nomogram diagnostic model in the validation cohort; (B) The ROC curves of the nomogram diagnostic model in the validation cohort; (C) The decision curve analysis of the nomogram diagnostic model in the validation cohort. ROC: Receiver operating characteristic; AUC: Area under the curve; STB: Spinal tuberculosis.

To validate our model’s efficiency, a calibration curve (Figure 6A) and an ROC curve (Figure 6B) were generated based on the training group, resulting in an area under the curve (AUC) value of 0.841. The diagnostic model demonstrated a C-value of 0.84 in the training group. The calibration curves demonstrated a high concordance between the nomogram-predicted values and the actual measurements. As depicted in the decision curve (Figure 6C), when the model’s threshold was set between 2% and 88%, it surpassed both the “all” and “none” lines, signifying the model’s clinical utility within the current context. Finally, for internal model validation, we employed the validation cohort, with corresponding calibration and ROC curves shown in Figure 7A and 7B, respectively. Both AUC and C-values stood at 0.83. The calibration curves demonstrated a high concordance between the nomogram-predicted values and the actual measurements. Therefore, our model displayed good clinical efficacy, as indicated by the decision curve in Figure 7C.

## Discussion

STB and PS represent the most prevalent forms of spinal infectious diseases [16, 17]. These two conditions often have overlapping clinical manifestations, with both exhibiting bony destruction, sclerotic bone changes surrounding the lesions, necrotic bone, and paraspinal abscesses evident on imaging studies [18]. Consequently, differentiating between STB and PS poses a significant challenge to healthcare professionals, with direct impacts on clinical decision making. Although tissue culture from the lesion site is considered the gold standard for diagnosing STB [19, 20], it is associated with certain limitations. Firstly, its sensitivity is not very high [19]. Secondly, obtaining tissue culture samples involves invasive procedures or open surgery. Furthermore, the culturing process is time consuming, frequently leading to diagnostic delays [3]. Such diagnostic delays can result in severe complications, as well as negatively affect patients’ quality of life [21]. Thus, there is a pressing need for the development of a rapid and non-invasive diagnostic model to differentiate between STB and PS.

Several previous studies have explored the utility of blood parameters in diagnosing TB, sparking the interest of numerous researchers. These studies have reported that routine laboratory indicators, including routine blood tests and biochemical tests, can provide valuable diagnostic insights for TB [22–24]. For instance, Chen et al. [25] identified the MLR as an independent diagnostic marker for STB, highlighting its correlation with STB severity. ML methods, which can efficiently analyze extensive datasets and identify patterns [26], have been applied to predict a range of medical conditions, including cardiovascular diseases [27], neonatal diseases [28], kidney diseases [29], and diabetes [30]. In our study, ML approaches were employed to identify seven factors associated with the diagnosis of STB and PS, and those were as follows: PNR, NLR, PDW, MPV, HGB, RBC, and age.

The NLR, calculated as the ratio of neutrophils to lymphocytes in peripheral blood, is emerging as a significant marker for various inflammatory diseases [31–33]. Our findings demonstrated that the NLR was lower in STB patients compared to PS patients, suggesting its potential as a diagnostic indicator in differentiating between the two conditions. This observation aligns with the results of Liu et al. [34]. Such difference can be attributed to the distinct immune responses induced by *Mycobacterium tuberculosis* in STB, which typically result in higher LYM and lower NEU in STB patients. In contrast, PS, a bacterial infection, typically triggers a more acute and neutrophil-dominant inflammatory response, thus resulting in elevated NLR levels in affected individuals. Nevertheless, it is worth noting that factors like the differences in patient demographics, disease stage at the time of diagnosis, and host immune response variations may influence the NLR level disparities.

Blood test findings in some TB patients frequently reveal thrombocytopenia [35] and neutrophil-dominant leukocytosis [36]. TB granulomas’ crucial constituents include neutrophils, macrophages, and lymphocytes. Necrotic granulomas house a significant number of neutrophils and eosinophils [37]. Our research indicates that a lower PNR is associated with a higher STB risk score, aligning with the previously mentioned results.

PDW measures the variability in platelet volume. Its role in the pathogenesis of TB is not clearly defined. Our study reveals that higher PDW values are associated with an increased likelihood of an STB diagnosis. This elevation might be a consequence of the inflammatory stimulus of STB, leading to reactive thrombocytosis and, subsequently, a rise in PDW. MPV serves as an inflammatory marker for various diseases [38]. TB influences MPV mainly through immune system alterations. It prompts inflammation and triggers an immune response, culminating in enhanced PLT production and, consequently, an increase in platelet volume. Activation of the immune system can induce inflammation, potentially affecting PLTs. Some studies suggest that chronic inflammation conditions may lead to reduced PLTs [39], potentially due to the cytokines and chemical mediators released by the immune system. These agents can influence platelet production and lifespan in the bone marrow. Currently, conclusive evidence linking TB directly to platelet alterations, or elucidating the precise role of the immune system, remains elusive [40].

The roles of HGB and RBC count in differentiating STB from PS are not fully elucidated. Certain studies suggest that the HGB and RBC levels decrease in cases of TB and suppurative inflammation [41, 42]. Conversely, HGB and RBC counts are observed to be significantly higher in STB patients compared to those with PS. The authors attribute the elevated HGB and RBC levels in STB patients to lower bodily consumption caused by the STB infection compared to PS. However, it is worth noting that changes in HGB and RBC counts lack specificity and can be affected by other factors. Therefore, when distinguishing between STB and PS, it is essential to consider various factors, including clinical symptoms, imaging findings, and pathogen detection.

An epidemiological study by Garg et al., encompassing 1652 STB patients, reported that the most prevalent age group for STB cases was 21–30 years (33%). Ages within the cohort ranged from 4 to 87 years, with a median of 32.4 years. Elderly patients (≥ 65 years) constituted a mere 4.6% of the sampled population [43]. The incidence of STB notably declined in adults above 40 years, corroborating the findings from our diagnostic model, which suggests that younger patients are at a higher risk of STB diagnosis. Several factors contribute to this trend. The typical younger age of STB patients may result from differences in disease etiology, transmission patterns, varying immune responses across age groups, and age-related risk factors. STB, typically caused by *Mycobacterium tuberculosis*, may have a higher transmission rate among younger individuals. Younger patients may also mount a stronger immune response. In contrast, PS, primarily bacterial in origin, might be more prevalent among older individuals, possibly due to age-related factors and comorbidities. Additionally, delayed diagnosis of STB in younger patients may also contribute to this age difference. In sum, the age discrepancy between these two conditions is likely multifaceted, influenced by variances in pathophysiology, immune responses, and risk factors. Further research is warranted to comprehensively investigate these factors.

In our research, we primarily focused on parameters derived from routine complete blood counts and utilized ML techniques to construct a valuable diagnostic model. This diagnostic model holds significant importance for several reasons. Firstly, it can play a critical role in diagnosis, offering valuable guidance before undertaking invasive procedures. Secondly, given that blood routine data is standardly collected in clinical assessments, acquiring this data is a straightforward and cost-effective process. Moreover, relevant parameter ratios can be easily calculated from the complete blood count data.

Our study underscores the clear advantages of utilizing ML in data analysis. ML excels in managing extensive and intricate datasets, identifying patterns, and extracting insights that may be challenging to discern using traditional statistical methods. Its capacity to adapt and self-improve based on data leads to more accurate predictions and classifications. This makes ML a potent tool in healthcare research, diagnostics, and decision making.

Nonetheless, this study exhibits certain limitations. Firstly, it is a one-arm study with an insufficient sample size, which could introduce both selection and subjective biases. Secondly, our study lacks external validation, which is necessary for evaluating the diagnostic model’s performance. Addressing these issues will require future multicenter studies with larger sample sizes. Thirdly, data related to fever presence, anti-TB antibody testing, T-Spot testing, or tuberculin testing were not collected. This omission arises from the fact that many PS patients at our hospital did not undergo TB-related evaluations, which could introduce potential biases in ML results. Finally, we did not incorporate data on patients’ medication histories due to limitations in data collection. Going forward, we aim to include a comprehensive examination of medication usage in subsequent research efforts.

## Conclusion

In conclusion, this study employed multiple ML algorithms to construct and validate a non-invasive nomogram diagnostic model, demonstrating robust diagnostic performance in differentiating between STB and PS. This model can facilitate clinical decision making, enabling healthcare professionals to make more accurate and prompt assessments.

## Supplemental data

**Table S1.** Clinical data of patients in the training cohort

Available at the following link:

https://docs.google.com/spreadsheets/d/1MGglyG91O5lQeEWaQ-WC96-OAMsHpvB-/edit?usp=sharing&ouid=101582347833441368642&rtpof=true&sd=true

**Table S2.** Clinical data of patients in the validation cohort

Available at the following link:

https://docs.google.com/spreadsheets/d/1PLbxR8Km-ySagOXFb2vS8ymrYrtffdfa/edit?usp=sharing&ouid=101582347833441368642&rtpof=true&sd=true

## Data Availability

The original contributions presented in this study are included in the article and its supplementary material. Further inquiries can be directed to the corresponding author.
